# Facial nerve paralysis caused by a T‐cell lymphoma

**DOI:** 10.1002/ccr3.3285

**Published:** 2020-09-15

**Authors:** Lisa Nachtsheim, Maike Wittersheim, Stefanie Kreissl, Jens Peter Klussmann, Maria Grosheva

**Affiliations:** ^1^ Department of Otorhinolaryngology, Head and Neck Surgery Medical Faculty University of Cologne Cologne Germany; ^2^ Department of Pathology Medical Faculty University of Cologne Cologne Germany; ^3^ Department I of Internal Medicine and Center of Integrated Oncology Medical Faculty University of Cologne Cologne Germany

## Abstract

Facial nerve paralysis due to the infiltration by a lymphoma is rare and the prognosis remains poor. If perineural spread and meningeosis are suspected, quick interdisciplinary diagnostic work‐up is recommended. It should include magnetic resonance imaging, biopsy of the lesion, bone marrow biopsy, and lumbar puncture. Therapy should be initiated immediately.

## Graphical abstract

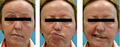

## 1 CASE HISTORY

**Figure 1:**
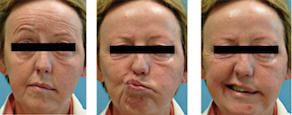
Peripheral complete flaccid facial nerve paralysis at admission

A 66‐year‐old female patient was admitted to our department with an acute complete peripheral facial nerve paralysis and pain of the left face, which had been present for several days (Figure 1). At admission, two skin lesions were present. Medical history revealed that previous biopsies of the same skin lesions, which had been taken a year ago in an external clinic, had been diagnosed as benign lymphomatoid papulosis. Therefore, the patient had been treated with local radiotherapy (2x4 Gy) and low dose methotrexate (MTX). The patient was still receiving a low maintenance dose of MTX (15 mg weekly). Furthermore, therapy for the trigeminal pain with pregabalin (200 mg) and prednisolone 50 mg for the facial palsy had already been initiated in the external neurological department.

## 2 EXAMINATION

**Figure 2:**
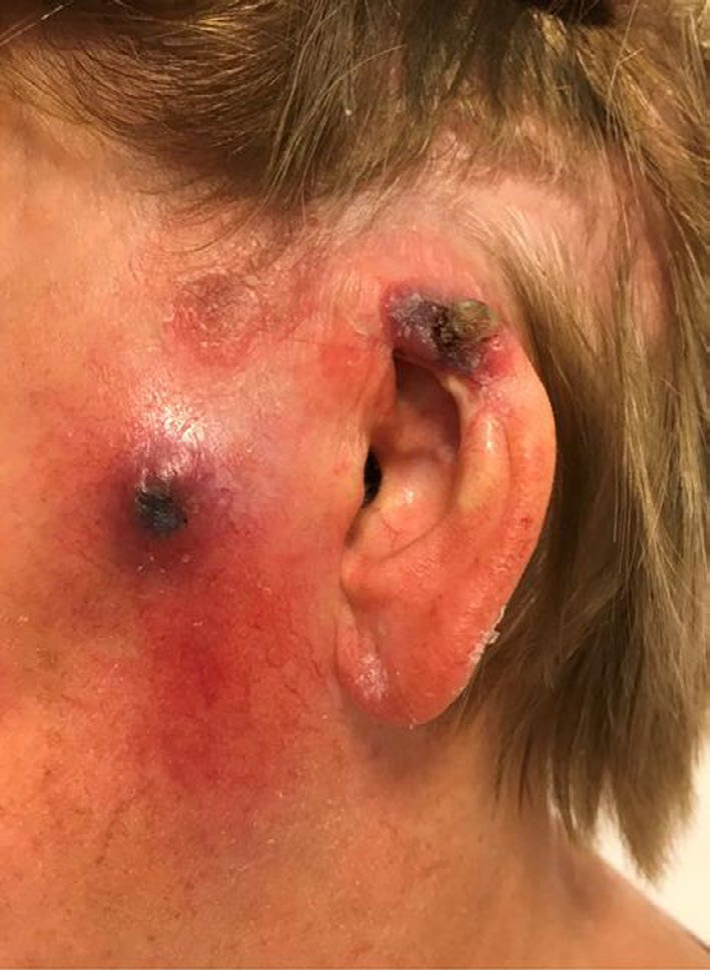
Skin lesions of the left auricle and cheek at admission

**Figure 3:**
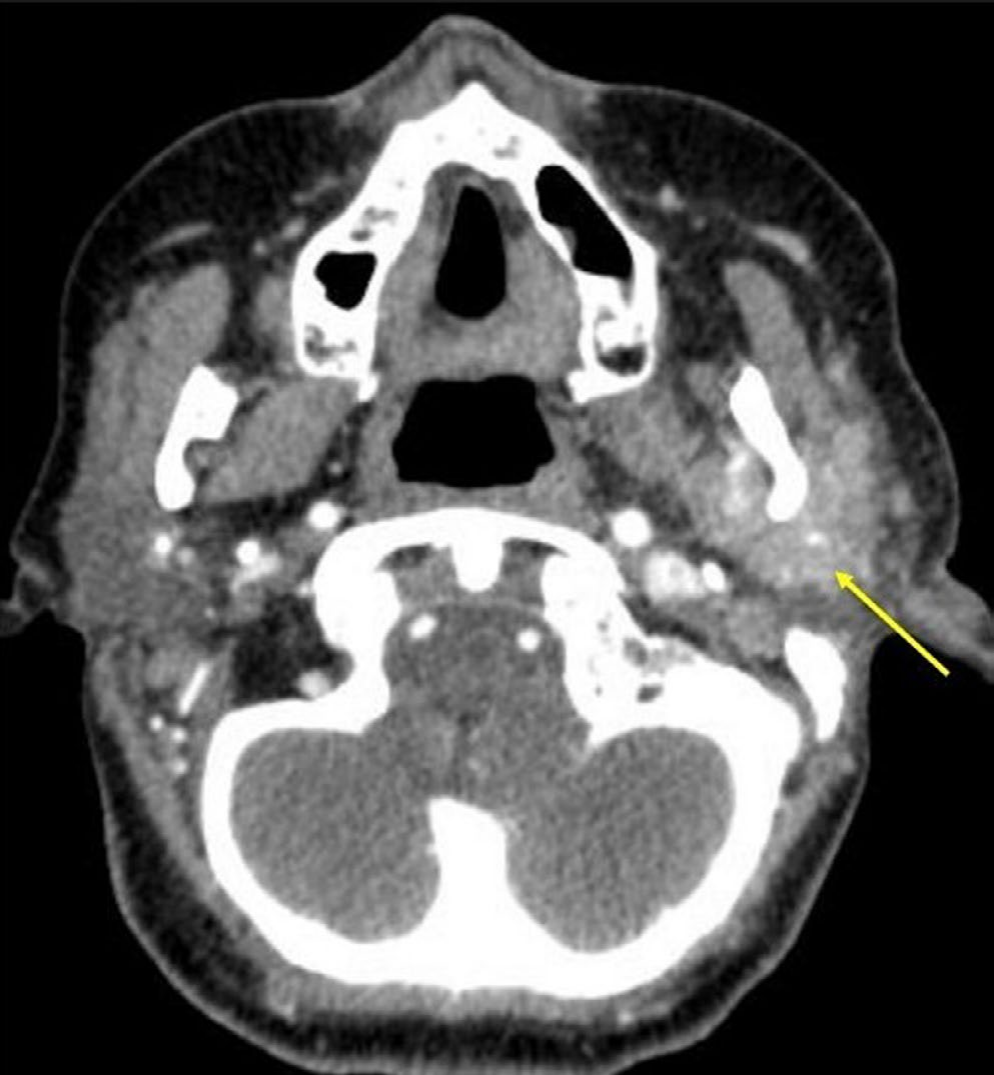
Axial CT scan with contrast. The arrow points toward the parotid lesion on the left side

At admission, suspicious skin lesions of the left ear and cheek were obvious (Figure 2). During ENT‐examination, the intraparotideal tumor was not palpable. However, a parotid lesion on the left side was evident in ultrasound and was confirmed in a CT scan with contrast (Figure 3). Serum analysis for neurotropic viral and bacterial infections was negative. The inner ear function was normal. Trigeminal trigger points (V1‐3) of the left face were hypersensitive and painful. The patient graded the pain level on the numeric analogous scale (NAS 0‐10) as NAS 5‐6. The patient showed a complete flaccid peripheral facial palsy with the House Brackmann Score VI.

## 3 DIFFERENTIAL DIAGNOSIS, INVESTIGATION

**Figure 4:**
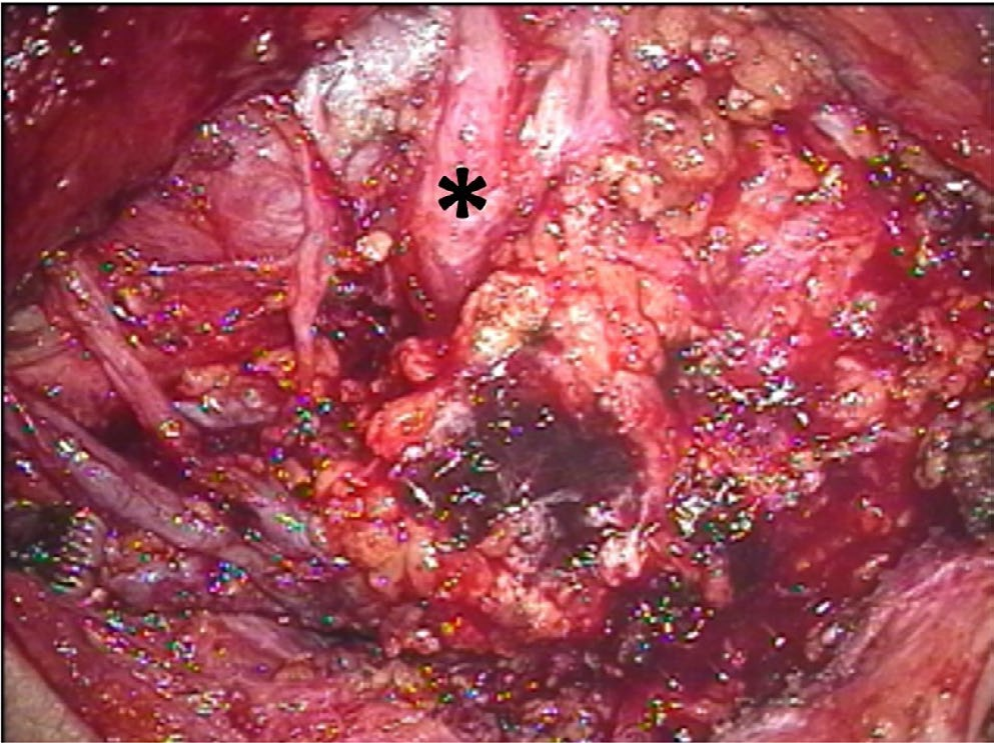
Intraoperative findings: Infiltration of the peripheral facial nerve trunk and a consequent edema of the peripheral branches (* marks the buccal branch) by a solid tumor

**Figure 5:**
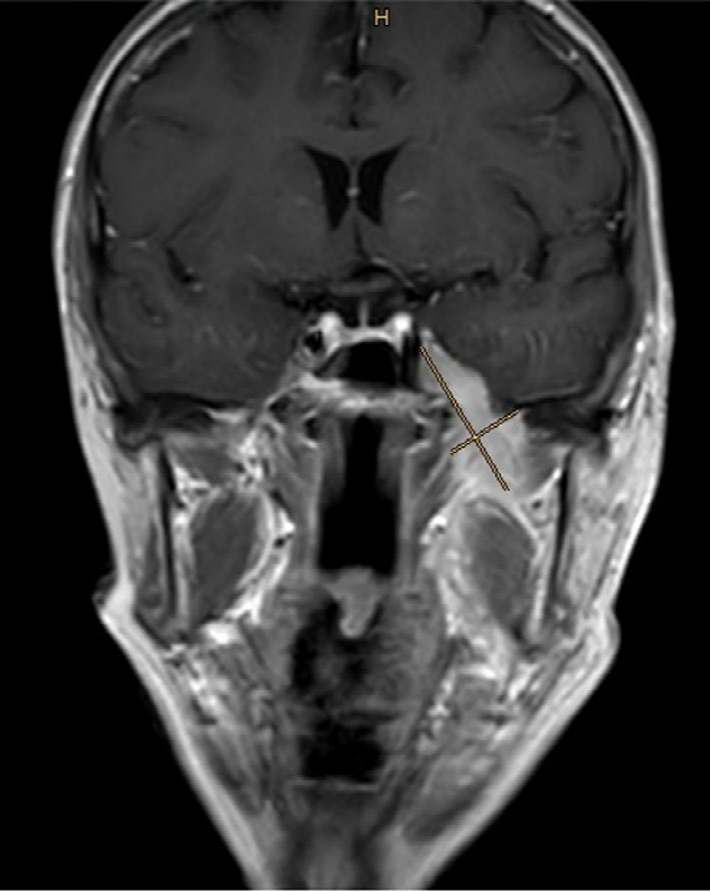
HE staining and CD 3 immunohistochemistry. Circumscribed infiltrates of the tumor next to regular salivary gland tissue. Tumor cells are strongly positive for CD3

**Figure 6:**
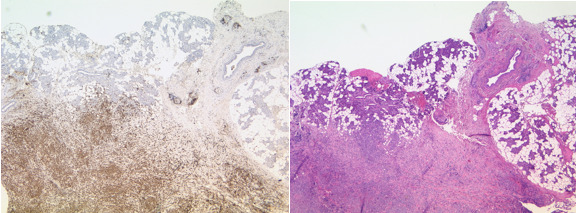
Intracranial expansion of the tumor due to perineural infiltration of the trigeminal nerve (4 × 1.8 cm) in a coronar T1‐MRI with contrast

First, we performed excisional biopsies of both skin lesions to exclude squamous skin cancer from diagnosis. Additionally, a fine‐needle aspiration cytology of the parotid lesion was carried out. The excisional biopsies of the skin lesions confirmed the presence of the lymphomatoid papulosis. The histological results of the fine‐needle aspiration cytology showed only sparse cells and no indication of malignancy. Because of unclear histology of the parotid tumor, we scheduled a diagnostic explorative parotidectomy and planned the intraoperative frozen section analysis. The patient was informed that in case of a solid malignant tumor (ie, salivary gland tumor or metastasis of the squamous cell cancer or melanoma), the extension of the surgery to a total or radical parotidectomy with reconstruction of the facial nerve was possible. During surgery, diffuse infiltration of the parotid tissue and the peripheral facial nerve was evident (Figure 4). Multiple biopsies of the surrounding parotid tissue were sent for immediate frozen section analysis because of the clinical suspicion of malignancy. The pathologist suspected infiltration of the parotid tissue by a malignant lymphoma, so that after the operation was aborted. The final immunohistochemical result confirmed the presence of the peripheral T‐cell lymphoma not otherwise specified (PTCL‐NOS; Figure 5). Because of clinically present diffuse infiltration of the facial nerve and persisting facial pain, an MRI scan was carried out to exclude perineural tumor spread, lymphangiosis, and other CNS involvement (Figure 6). In the MRI scan of the head, perineural spread of the lymphoma along the trigeminal and facial nerve and consecutive carcinomatous meningeosis was present. Furthermore, lumbar puncture showed infiltration of the lymphoma in the cerebrospinal fluid. The patient was immediately referred to oncology department for initiation of the treatment.

## 4 TREATMENT

There is currently no established standard treatment for PTCL‐NOS, but high dose chemotherapy following autologous stem cell transplantation showed good treatment outcomes in several trials.^1^ In our oncology department, the patient received a treatment similar to most CNS lymphomas following the MATRIx regime, excluding rituximab.^2^ The therapy was personalized and consisted of four cycles (10 weeks) high dose MTX (500 mg/m^2^), Ara‐C (2000 mg/m^2^), and thiotepa (30 mg/m^2^), which were followed by high dose chemotherapy (carmustin 490 mg/m^2^ and thiotepa 5 mg/kg) and autologous stem cell transplantation.

## 5 OUTCOME AND FOLLOW‐UP

The patient showed partial response to the treatment after the initial chemotherapy because of persisting infiltration of the lymphoma in the cerebrospinal fluid. Restaging including an MRI of the head showed a regredient contrast enhancement in the residual findings in the left parotideal area. However, the peripheral complete facial paralysis was still present after 3 months. No voluntary movement was seen. In the needle electromyography, sporadic spontaneous activity was present in the frontalis muscle at rest. Besides, no muscle action potentials were present during voluntary movement of the main facial muscles. The patient received logopaedic therapy to relieve the hypertension of the healthy side of the face. Furthermore, a tarsorraphy of the lower lid was performed by the ophthalmologists. Because of persisting carcinomatous meningeosis in the lumbar puncture 2 months after stem cell transplantation, a weekly intrathecal treatment with MTX 15 mg, cytarabin 40 mg, and dexamethason 4 mg was initiated. Due to continuing progress of the carcinomatous meningeosis, radiation therapy of the CNS was initiated. The patient died 8 months after the initial diagnosis of progressive disease.

## 6 DISCUSSION

Manifestation of a lymphoma in the parotid gland and especially perineural infiltration of the facial nerve by a lymphoma is very rare. Only 10%‐15% of all malignant lymphomas present as peripheral T‐cell lymphomas.^1^ The PTCL‐NOS is a subtype of non‐Hodgkin lymphoma that develops from T cells and natural killer cells.^3^ PTCL‐NOS is characterized by its aggressive behavior and very poor prognosis.^4^ If perineural spread and meningeosis are suspected, quick interdisciplinary diagnostic work‐up is recommended. It should include MRI, biopsy of the lesion(s), bone marrow biopsy, and lumbar puncture. If the diagnosis of a T‐cell lymphoma is confirmed, the oncologic therapy should be initiated immediately although standard treatment and outcome still remain poor.^5^ The 5‐year overall survival rate is 20%‐30%.^6^

Lymphoma cells carry a steroid receptor which triggers apoptosis and consequent cell lysis.^7^ If lymphoma is suspected, corticosteroid treatment (ie, for facial nerve palsy) should be admitted very carefully, because it can change cellular morphology and thus delay and complicate the histological diagnosis.^8^

## CONFLICT OF INTEREST

There was no conflict of interest during the preparation of this article. The patient’s family approved the publication of this article.

## AUTHOR CONTRIBUTIONS

Authors Lisa Nachtsheim, Jens Peter Klussmann, and Maria Grosheva have made substantial contributions to conception and design of the manuscript, and to the interpretation of the data. They were involved in drafting the manuscript. Maike Wittersheim is a specialist in pathology and contributed substantially to the interpretation of the data. Stefanie Kreissl is a specialist in clinical oncology and contributed substantially to the therapy and the follow‐up sections of the manuscript. All authors were involved in revising the manuscript critically for important intellectual content; and have given final approval of the current version to be published. All authors have agreed to be accountable for all aspects of the work in ensuring that questions related to the accuracy or integrity of any part of the work are appropriately investigated and resolved.

## References

[1] Yamaguchi M , Suzuki R . JSH practical guidelines for hematological malignancies, 2018: 7. Peripheral T‐cell lymphoma (PTCL). Int J Hematol. 2019;109(2):137‐140. 30671910 10.1007/s12185-018-02589-4

[2] Ferreri AJM . Therapy of primary CNS lymphoma: role of intensity, radiation, and novel agents. Hematology. 2017;2017(1):565‐577. 29222306 10.1182/asheducation-2017.1.565 PMC6142584

[3] Swerdlow SH , Campo E , Harris NL , et al. WHO Classification of Tumours of Haematopoietic and Lymphoid Tissues. Lyon: IARC; 2008.

[4] Wang SS , Vose J . Epidemiology and prognosis of T‐cell lymphoma In Foss F (Ed.), T‐Cell Lymphomas. Springer 2013.

[5] Al‐Zahrani M , Savage KJ . Peripheral T‐cell lymphoma, not otherwise specified: a review of current disease understanding and therapeutic approaches. Hematol Oncol Clin North Am. 2017;31(2):189‐207. 28340873 10.1016/j.hoc.2016.11.009

[6] Oluwasanjo A , Kartan S , Johnson W , et al. Peripheral T‐cell lymphoma, not otherwise specified (PTCL‐NOS). Cancer Treat Res. 2019;176:83‐98. 30596214 10.1007/978-3-319-99716-2_4

[7] Weller M . Glucocorticoid treatment of primary CNS lymphoma. J Neurooncol. 1999;43:237‐239. 10563429 10.1023/a:1006254518848

[8] Borenstein S , Gerstle T , Malkin D , Thorner P , Filler R . The effects of prebiopsy corticosteroid treatment on the diagnosis of mediastinal lymphoma. J Pediatr Surg. 2000;35(6):973‐976. 10873047 10.1053/jpsu.2000.6945

